# Management of large congenital parameatal cyst: Observation or intervention? (Case Report)

**DOI:** 10.1016/j.ijscr.2020.03.028

**Published:** 2020-03-28

**Authors:** Christopher A. Christensen, Veronica Mugarab-Samedi

**Affiliations:** a Department of Pediatrics, Jim Pattison Children’s Hospital, Saskatoon S7K 1M6, Canada; b University of Saskatchewan, Saskatoon S7K 1M6, Canada

**Keywords:** Parameatal cyst, Neonate, Congenital

## Abstract

**Introduction:**

A parameatal urethral cyst is a rare finding in an otherwise well child. They can present as either congenital malformations or as a finding later in life, and may occur either unilaterally or bilaterally on the glans penis. Though they are benign, possible complications include impaired urinary stream flow, dysuria, or hindered cosmesis.

**Presentation of case:**

We report a spontaneous resolution of large congenital parameatal cyst in an otherwise healthy neonate whose mother had a recent history of urinary tract infection. A spontaneous resolution of the cyst was reported at the age of one month.

**Discussion:**

A small parameatal cysts have shown spontaneous resolution approximately 25% of the time. Treatment options should not include aspiration or marsupialization as many cases have shown recurrence. The role of antenatal infection in development of parameatal cyst not yet been determined.

**Conclusion:**

A large parameatal cyst could resolve spontaneously, routine observation recommended for majority of cases.

## Highlights

• A congenital parameatal cyst (CPM) can be concerning to parents.
• CPM usually presents before the onset of puberty, and is more common in males than females.
• Spontaneous resolution of CPM reported in few situations.
• The etiology of CPM is largely not understood.
• Association of CPM with maternal infection is uncommon.

## 1 Introduction

A congenital urethral parameatal cyst can be concerning to parents due to both their infrequent occurrence and distressing appearance. They present more commonly in males than females, and usually before the onset of puberty [1]. The etiology is largely not understood. When first described, the pathogenesis was believed to be due to preputial delamination and cyst development [2]. A separate view suggests the cyst originates from a blockage of the paraurethral duct [3] with suggestions that they may occur alongside an infectious process [4], though this is disputed [1]. Limited pathology reports available described the cysts as containing either singular or multiple components of transitional, cuboidal, or columnar epithelia with no clear source of origin [1]. We present a case of spontaneous resolution of large congenital parameatal cyst in an otherwise healthy neonate whose mother had a recent history of urinary tract infection. This manuscript has been reported in line with the SCARE criteria [5].

## 2 Case report

**Fig. 1:**
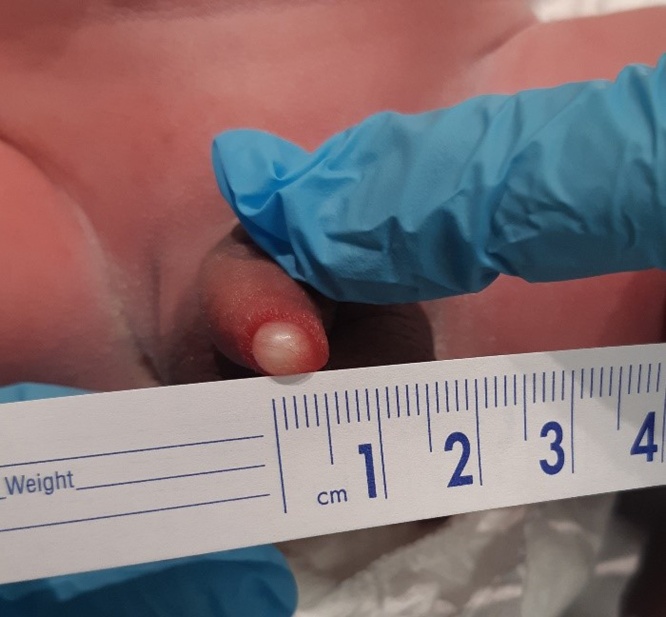
Congenital parameatal cyst measuring 5 mm in diameter.

**Fig. 2:**
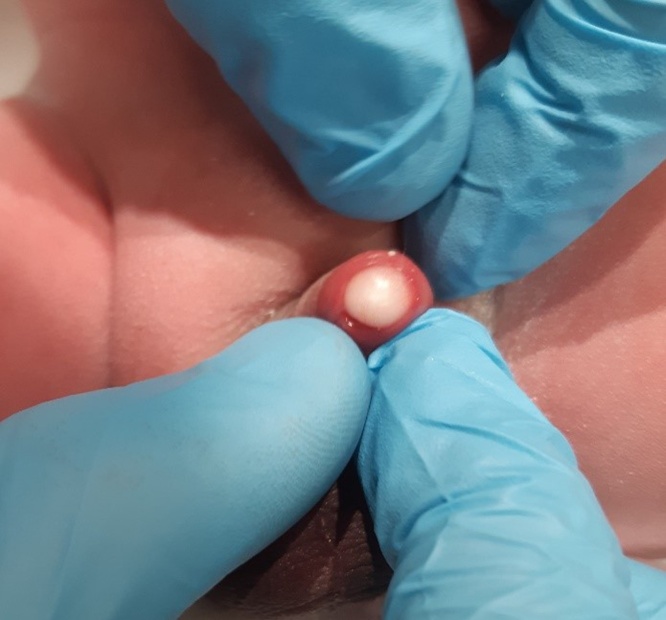
Viewpoint showing the frontal obstruction of the urethra.

**Fig. 3:**
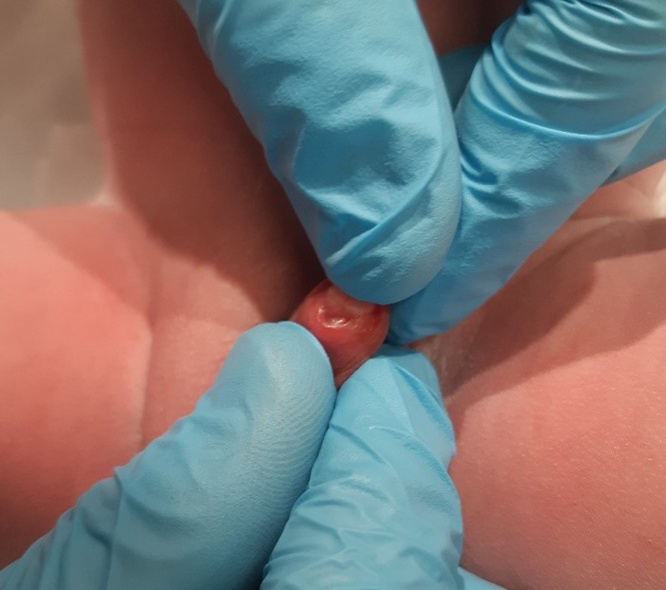
Movement of the sac away from the urethral opening.

The NICU team was called to assist in the delivery of a 35-year-old G3P2 woman, pregnant at 38 + 6 weeks in for repeat caesarean. Labour had started 5 h prior with rupture of membrane at delivery. There was no maternal fever and the amniotic fluid was not malodorous. Delivery was unremarkable, with stimulation being required for resuscitation and Apgar scores of 5, 9, and 9 (at 1, 5, and 10 min respectively). During the physical exam, a large 5 mm cyst was found on the left lateral side of urethral meatus with no associated hypospadias or other abnormalities. The sac was large enough to completely obstruct the view of the urethra even after gentle pressure was applied. Inspection of the sac showed a fluid-filled cavity containing an off-white thickened liquid. The sac itself was not easily separated from the tissue with light pressure, and was non-mobile and non-tender (Figs. 1, 2 and 3). The remainder of the physical exam was otherwise normal. Shortly after examination, the neonate was placed back into the care of his family where hours later, micturition occurred. Pediatric Urology was consulted and suggested conservative management. Renal ultrasound was performed prior to discharge home, and was reported as a normal. Follow up by family physician at 4 weeks of age revealed spontaneous resolution of the cyst.

On review of the obstetrical history, the mother had a urinary tract infection positive for E. faecalis and E. coli during week 34 of pregnancy which was treated with Cephalexin. This may contribute to view of a concomitant infection or inflammation affecting the development of a cyst.

## 3 Discussion

In the majority of cases, a small parameatal cyst is asymptomatic and does not require urgent interventions [1]. There are no known co-morbidities or syndromes associated with the condition. Previous cases have shown spontaneous resolution approximately 25% of the time [6], for which the only recommendation was routine observation. However, in a minority of patients, painful or difficult urination may result if the sac begins to obstruct the urethral passage. Sizes have been described ranging from 2 mm to 10 mm, which very rarely increases in size with age [6]. Physicians will most commonly be consulted for cosmetic reasons as the child begins to grow and the cyst remains unruptured.

Treatment options should not include aspiration or marsupialization as the described cases have shown recurrence or continuous drainage [7]. Surgical extraction of the entire sac has shown to be a reliable way of symptomatic management and has an excellent cosmetic prognosis.

It has not yet been determined if infection can contribute to a parameatal sac development, but interestingly the appearance of a large congenital cyst appeared in the context of a previous maternal UTI. Our patient was otherwise well and able to pass urine without difficulty, despite the size of the cyst. His urinalysis results were normal, with no signs of infection or inflammation. Renal ultrasound did not reveal any anomalies, and patient was discharged home. Follow-up with the family doctor was recommended to monitor for cyst evolution. No complications or associated symptoms were noted, patient was doing and growing well. A spontaneous resolution of the cyst was reported at the age of one month. As possible re-occurrence of parameatal cysts was reported in several cases [[6], [7], [8]], family physician will continue to follow the patient up to the age of 12 months.

## Declaration of Competing Interest

None declared.

## Sources of funding

Not funded.

## Ethical approval

As per University of Saskatchewan REB publication of case report doesn’t require ethical approval if researchers protect the right to anonymity and confidentiality of the patient.

As our patient is neonate parental consent to share history and photographs was obtained for this publication.

## Consent

Written informed consent obtained from the parents of the patient.

## Author contribution

Dr. Christiansen – patient care, data collection, literature review, manuscript preparation.

Dr. Veronica Mugarab-Samedi – manuscript edition, literature review, patient follow up.

## Registration of research studies

Not required.

## Guarantor

Dr. Veronica Mugarab-Samedi.

## Provenance and peer review

Editorially reviewed, not externally peer-reviewed.
